# Enhancing organizational resilience of emergency medical services through a designated intervention program

**DOI:** 10.1186/s13584-026-00772-1

**Published:** 2026-07-23

**Authors:** Tamir Velner, Zohar Rubinstein, Bruria Adini

**Affiliations:** 1 Emergency & Disaster Management Department, School of Public Health, Gray Faculty of Medical and Health Sciences, Tel Aviv University, Tel Aviv, 6139001 Israel; 2 Baruch Ivcher School of Psychology, Reichman University, Herzliya, Israel; 3 ResWell — Multi-National Research Collaboration on Resilience and Well-Being, Tel Aviv University, Tel Aviv, 6139001 Israel

**Keywords:** Organizational resilience, Emergency medical services, Resilience intervention program, Quasi-longitudinal study, Conflict-affected settings, Transformational leadership, Employee well-being, Technological advancement

## Abstract

**Background:**

Emergency medical services (EMS) in conflict-affected settings face compounding operational demands, making organizational resilience (OR) a critical institutional asset. Israel’s EMS have operated under ongoing security pressures for decades, intensified dramatically following the 7 October 2023 attacks. Despite growing recognition of OR as a measurable and modifiable property, longitudinal interventional evidence in pre-hospital settings remains sparse. This study reports the development and three-time-point evaluation of a targeted intervention program designed to enhance OR within Israeli EMS.

**Methods:**

A three-time-point quasi-longitudinal interventional study was conducted within a single EMS: T1 (pre-intervention baseline; *n* = 196), T2 (post-intervention; *n* = 205), and T3 (six-month follow-up; *n* = 172). OR was measured using the validated seven-domain Organizational Resilience Questionnaire (ORQ). The 70-hour intervention - comprising structured training (30 h, 4 weeks) and organizational embedding (40 h, 24 weeks) - was delivered by internal Resilience Trustees. OR changes were examined using one-way ANOVA with Bonferroni-corrected comparisons (Cohen’s d) and independent-samples t-tests (α*=0.005). Sequential multivariable regression (pooled *n* = 573) and ANCOVA identified independent OR predictors and examined OR changes after covariate adjustment.

**Results:**

Composite OR increased significantly from T1 to T2 (Δ=+0.20; *p* = 0.018; d = 0.27), with a Bonferroni-corrected significant gain in situational awareness (Δ=+0.25; *p* < 0.001), with additional gains in adaptive capacity (Δ=+0.21; *p* = 0.006) and learning processes (Δ=+0.23; *p* = 0.006) reaching conventional but not Bonferroni-corrected significance. OR continued to rise at six-month follow-up (T1 mean = 3.86; T2 mean = 4.06; T3 mean = 4.09; Δ T1→T3 = + 0.23; *p* = 0.007; d = 0.32). The time effect remained significant after full covariate adjustment (ANCOVA: F = 7.41; *p* = 0.001; ηp²=0.026), with paramedics showing greater gains (+ 0.27) than other staff (+ 0.05; Time × Profession: *p* = 0.046). Transformational leadership, employee well-being, and technological advancement were the dominant OR predictors (pooled R²=0.770; β = 0.458, 0.274, 0.208, respectively; all *p* < 0.001).

**Conclusions:**

A structured, domain-targeted OR program was associated with significant, domain-coherent resilience gains that progressively consolidated under protracted conflict conditions. The ORQ and the Resilience Trustee model — for which the present study provides preliminary effectiveness evidence — together offer a promising framework for OR strengthening in EMS organizations confronting chronic operational disruption, with implications for national and global emergency health policy.

**Supplementary Information:**

The online version contains supplementary material available at 10.1186/s13584-026-00772-1.

## Background

Emergency medical services (EMS) operate at the intersection of acute clinical demand and public security, required to sustain continuity when systemic pressures are highest. In Israel, this exposure is ongoing: EMS have operated under a persistent security threat for decades, with the 7 October 2023 attacks and the protracted conflict that followed representing the most severe escalation to date, testing pre-hospital services under conditions of simultaneous high-casualty volume, staff’s personal risk, and systemic uncertainty [1, 2].

Israel’s emergency health preparedness system is grounded in multiple legislative frameworks, including the Public Health Ordinance (1940) and the Civil Defense Law (1951), with the Ministry of Health’s Supreme Health Authority coordinating national organizational readiness across the health system [3]. The national EMS operates across geographically diverse regions with distinct operational, demographic, and security profiles. The combination of a technologically advanced health system, a well-developed emergency preparedness infrastructure, and continuous conflict-related operational stress constitutes a demanding yet highly informative context for OR research [4, 5], with direct relevance to emergency services operating in other conflict-affected or high-risk environments [1, 4, 5, 6, 7].

Organizational resilience (OR) is the capacity of an organization to anticipate, prepare for, absorb, and adapt to a changing environment; to cope effectively with adverse events while maintaining functional continuity; and to recover quickly and efficiently. This definition synthesizes anticipatory, absorptive, adaptive, and recovery dimensions [8, 9, 10, 11] and positions OR as an organizational-level property amenable to structured measurement and targeted intervention [10, 11].

Despite growing theoretical interest in OR, rigorous interventional evidence at the organizational level is limited, particularly in pre-hospital settings [12, 13]. Systematic reviews consistently note that most existing programs target individual staff resilience rather than OR as an institutional capacity [13, 14, 15], and that multi-level programs combining training, coaching, and governance-level embedding are better suited to producing sustained change [16, 17]. A widely cited scoping review identified over thirty OR measurement tools but documented few controlled, organizational-level intervention studies; the most relevant recent frameworks draw on public-sector resilience programs and multi-level coaching models targeting healthcare managers [13, 18].

The present study addresses this gap by reporting on the development, implementation, and three-time-point empirical evaluation of a targeted OR intervention within an Israeli EMS.

The primary aim of the study was to examine OR changes associated with a targeted organizational resilience (OR) intervention program over time within an EMS. The secondary aim was to identify independent organizational predictors of OR and quantify their relative contributions within a pooled multivariable framework incorporating observations across all three measurements.

Three hypotheses were specified. H1: OR will increase significantly from T1 (pre-intervention baseline) to T2 (immediate post-intervention assessment), suggesting a short-term effect. H2: The OR gains observed at T2 will be sustained at T3 (six-month follow-up), with OR remaining significantly above the T1 pre-intervention baseline, thereby providing evidence regarding the durability of the intervention effects. H3: Transformational leadership, employee well-being, and technological advancement will each emerge as positive, significant, and independent predictors of OR in a pooled multivariable model incorporating data from all three measurement time points.

## Methods

### Study design and setting

A three-time-point quasi-longitudinal interventional study using repeated cross-sectional surveys was conducted within an EMS operating in southern Israel, selected for its operational relevance, sustained conflict-related pressures, and organizational capacity to support a structured programmatic evaluation. Three time-point measurements were defined: T1 (pre-intervention baseline; January–February 2024), T2 (post-intervention assessment; May–June 2025), and T3 (six-month follow-up; January–February 2026). The findings of the T0 pilot study were reported in a previous article [19]. No participant-level data from the T0 pilot study were included in the present analyses. The datasets analyzed in this manuscript comprise only the three repeated cross-sectional surveys conducted at T1 (2024), T2 (2025), and T3 (2026).

The intervention was delivered in two phases: Phase I (structured training; 30 h) was conducted over four weeks from November to December 2024, and Phase II (organizational embedding; 40 h) commenced immediately thereafter and was completed in May 2025, following 24 weeks of structured Resilience Trustee activities within the intervention district. T2 was administered immediately upon Phase II completion, establishing it as the primary post-intervention assessment point, and T3 was conducted approximately six months thereafter, enabling examination of the durability of OR changes over time.

### Participants and recruitment

Eligible participants were emergency rescue professionals and administrative staff employed or volunteering within the EMS at each measurement occasion. Recruitment was conducted via internal organizational mailing lists, a secure web-based platform, and formal communication channels. Participation was voluntary and anonymous. Of the approximately 1,100 employees comprising the intervention district, the questionnaire was distributed at each measurement occasion to an eligible sampling frame of approximately 600 staff members — those employed at a minimum of 25% of a full-time-equivalent position and with at least 18 months of organizational tenure at the time of administration; the corresponding response rates ranged from approximately 28% to 34% across the three waves. Sample size adequacy was determined with reference to parameters established in the ORQ validation study [20]: using G*Power software for a multivariate linear regression model with 10 predictors (f² = 0.15; α = 0.05; 1–β = 0.95), the minimum required sample size was 166 participants per measurement occasion. All three cohorts in the present study exceeded this threshold (T1: *n* = 196; T2: *n* = 205; T3: *n* = 172). These represent the final valid analytic samples retained after a pre-specified screening procedure. Eligibility for analysis required completion of at least 95% of questionnaire items. Within the principal study variables — organizational resilience and its seven constituent domains, transformational leadership, technological advancement, and employee well-being — a single omitted item was resolved by computing the respondent’s score for that variable from the completed items, whereas a questionnaire with two or more omitted items within any such variable was deemed invalid and excluded from analysis. Missing responses to individual demographic items did not invalidate a questionnaire, provided the 95% completion criterion was met; the respondent concerned was omitted only from analyses involving that specific demographic variable. Because residual item-level missingness was minimal and resolved a priori under these rules, a formal model-based assessment of the missing-data mechanism (MCAR/MAR/MNAR) was not undertaken.

A post-hoc power analysis was conducted using G*Power (independent-samples t-test) to confirm sample adequacy for the primary between-time-point comparison. Based on the observed T1–T3 effect size (d = 0.32), with α = 0.05 and *n* = 172 at T3, achieved power was 0.83, exceeding the conventional 0.80 threshold. The study was therefore adequately powered for the primary temporal comparison, complementing the a priori sample size calculation conducted for the multivariable regression model.

### Measures

#### Organizational resilience

OR was measured with the Organizational Resilience Questionnaire (ORQ) [20], a validated 29-item instrument spanning seven domains: Leadership and Human Capital (2 items), Organizational Culture (4 items), Situational Awareness (7 items), Adaptive Capacity (7 items), Learning Processes (3 items), Ethos (3 items), and Organizational Performance (3 items). Items are rated on a five-point Likert-type scale (1 = strongly disagree to 5 = strongly agree), yielding both domain-level and composite OR scores. The ORQ was developed and validated using a multi-organization healthcare sample (*n* = 602) in southern Israel, incorporating exploratory and confirmatory factor analysis, expert content validity assessment (12 experts), and an adapted Delphi process (19 experts in the first round, 16 in the second; consensus threshold ≥ 75%) to establish validated domain-weight rankings (Leadership and Human Capital 19%, Adaptive Capacity 18%, Organizational Culture 16%, Situational Awareness 13%, Learning Processes 13%, Ethos 11%, Organizational Performance 11%). Composite Cronbach’s α ranged from 0.96 to 0.97 across the three participating healthcare organizations at the baseline measurement (T0), reflecting the reliability of the full composite score encompassing all seven ORQ domains.

ORQ development commenced in 2022 as part of the study underpinning the present research, beginning with a systematic review of the organizational resilience literature to identify and operationalize domain-level constructs — a stage that preceded the Delphi process and pilot administration described above [20]; the validation process itself predates T1 data collection (January–February 2024) by approximately two years (the 2026 publication date of Ref. 20 reflects the date of publication rather than the timing of the validation). The T0 pilot data served exclusively to inform program domain prioritization and instrument refinement and were not included in any primary analyses reported in this manuscript. Accordingly, there is no analytical overlap between the pilot-study dataset and the datasets reported in the present manuscript.

#### Organizational predictors and covariates

Three organizational-level variables - transformational leadership, employee well-being, and technological advancement - were included as independent predictors on the basis of their theoretical centrality in the resilience and emergency services literature. Transformational leadership was measured using the Leadership Quotient (LQ) Model [19, 21], a validated 9-item instrument capturing leadership dimensions including vision, empowerment, and alignment with organizational values (Cronbach’s α = 0.93). Technological advancement was assessed using a validated 7-item scale developed by Chen and Huang [19, 22], reflecting managerial and technical innovation (α = 0.94). Employee well-being was measured using the Workplace Well-Being Scale [19, 23], a 9-item instrument evaluating perceptions of job security, organizational support, professional growth, and workplace climate (α = 0.87). All three instruments were translated into Hebrew using forward–backward procedures in accordance with Brislin’s cross-cultural adaptation guidelines [24]. All three scales used the same five-point Likert-type response format as the ORQ. These variables also served a dual function: analytically, they were entered as predictors in multivariable regression and ANCOVA models; methodologically, their prior identification as key OR drivers during ORQ validation shaped the curriculum prioritization framework of the designated intervention program. Sociodemographic covariates — age, gender, professional role (paramedic vs. other), seniority, level of education, managerial status (manager vs. non-manager), religiosity, and income level — were included as independent variables.

### Intervention: development and architecture

#### Program development and domain prioritization

The OR intervention was developed through the systematic integration of four complementary phases, each contributing distinct and non-overlapping inputs to curriculum design and domain prioritization.

First, the pilot-study organizational baseline assessment (T0; 2022) provided empirical quantification of OR levels across all seven ORQ domains within three healthcare institutes, enabling evidence-based identification of the domains most in need of targeted strengthening.

Second, an adapted Delphi process was employed, through which a panel of content experts iteratively rated and refined the relative importance of each OR domain across successive rounds until a predefined consensus threshold was reached, thereby establishing a validated domain-weight hierarchy that directly informed curriculum emphasis [20].

Third, a systematic literature review was conducted encompassing OR theory and crisis management practice within emergency services contexts, ensuring alignment between program content and the current evidence base.

Fourth, structured consultations were conducted with specialists in organizational psychology with extensive experience in healthcare intervention design, providing practitioner-level validation of the proposed content and delivery format.

These steps were designed with the aim of maximizing the validity and effectiveness of the intervention program, and they preceded the pilot phase. Importantly, the implementation of the intervention within the EMS organization concurrently constituted the pilot phase of the program itself, such that the present study represents an organizational resilience intervention and the program’s inaugural real-world deployment simultaneously .

#### Resilience trustees

Senior EMS leaders formally designated Resilience Trustees - internal change agents, selected on the basis of positional influence, peer-change capacity, organizational credibility, and willingness to lead structured change. Each trustee was issued a written letter of appointment. This model is supported by implementation science evidence on embedded organizational champions [25]. Each trustee operates according to a structured work plan containing time-bound tasks and organizational resilience milestones, thereby propagating change progressively across the organization over time. A central design feature of this approach is the development of an enduring internal organizational capability: once trained, the Resilience Trustees carry forward the OR mandate autonomously, embedding resilience practices into the organization’s routine functioning across stations and units over the long term without reliance on recurring external training or consultancy.

Twenty Resilience Trustees were drawn from all five operational stations in the intervention district — four per station — ensuring broad and representative organizational coverage. All 20 Trustees completed both Phase I and Phase II in their entirety, with no attrition throughout the intervention. The program followed a ripple-effect logic: trustees served as embedded change agents, disseminating resilience competencies among station staff during Phase II, with the expectation that resilience-relevant norms, behaviors, and organizational practices would diffuse progressively across the broader workforce. Population-level ORQ measurement at T1, T2, and T3 was accordingly designed to capture organizational-level change, consistent with the validated purpose of the ORQ as an organizational rather than individual resilience measure [20]. All survey respondents at T1, T2, and T3 were members of the intervention district and were accordingly exposed to the organizational resilience strengthening project at the district level through operational awareness activities and active participation in selected program components. Individual-level exposure data — distinguishing between full, partial, and minimal engagement — were not systematically collected.

The Resilience Trustees operated within a bi-directional change strategy: bottom-up translation of program content into unit-level routines, and top-down engagement of senior leadership to secure executive commitment. The intervention included two main phases.

#### Phase I: structured training (30 h, 4 weeks)

Thirty hours of in-organization training were delivered over four full-day weekly sessions (~ 8 h each), with each session covering two thematic modules. Session 1 (Week 1; ~5 h): Program Orientation and Ethos. Session 2 (Week 2; ~8 h): Organizational Culture and Leadership and Human Capital. Session 3 (Week 3; ~10 h): Situational Awareness and Adaptive Capacity (scenario-based simulation). Session 4 (Week 4; ~7 h): Learning Processes and Organizational Performance. The trustees developed an organizational action plan upon completion of phase I.

#### Phase II: organizational embedding (40 h, 24 weeks)

Forty hours of coaching, structured review, and organizational embedding followed over 24 weeks (eight sessions, every three weeks, conducted in-person or via Zoom). Core activities included unit-level OR gap analysis, co-design of domain-specific micro-interventions, reflection sessions, lessons-learned documentation, steering committee reporting, and cross-unit coordination.

Fidelity across units during Phase II was maintained through four mechanisms: (a) each Resilience Trustee’s structured work plan incorporated domain-specific milestones against which progress was formally tracked; (b) the regular group coaching sessions served as the primary fidelity monitoring venue, with particular engagement from the five station managers in the intervention district; (c) each trustee submitted an interim progress report to the steering committee at weeks 12 and 24; and (d) the seven-domain ORQ framework functioned as a shared organizing template across all units. Residual inter-unit variation in implementation depth is inherent to any distributed change-agent model and is acknowledged in the Limitations.

Table 1 presents the full program architecture.

**Table 1:** Organizational resilience intervention program: structure, content, and expected outcomes

Component	Phase and duration	OR domain(s) (weight)	Key activities	Expected organizational outcome
**Phase I: Structured training − 30 h over 4 weeks (one full-day session per week; 2 content modules per session)**				
Session 1: Program orientation	Training; 2 h	Organizational Performance (11%); Leadership & Human Capital (19%)	OR framework introduction; Trustee governance role; program rationale; unit-level accountability orientation	Shared OR framework understanding; trustee role internalization; commitment to seven-domain architecture
Session 2: Ethos & meaning-making	Training; 3 h	Ethos & Meaning-Making (11%)	Values clarification; mission coherence workshops; shared purpose exercises; sense-making under adversity	Strengthened mission identity; enhanced crisis sense-making capacity; improved staff commitment
Session 3: Organizational culture	Training; 4 h	Organizational Culture (16%)	Culture mapping; psychological safety enhancement; trust-building; inter-unit collaboration norms; peer support	Improved psychological safety climate; stronger cross-unit cooperation and organizational social capital
Session 4: Leadership & human capital	Training; 4 h	Leadership & Human Capital (19%) - highest weight	Transformational leadership development; distributed decision-making; workforce competency mapping; succession planning	Strengthened leadership practices; enhanced workforce capability; improved distributed decision-making
Session 5: Situational awareness	Training; 4 h	Situational Awareness (13%)	Information architecture design; environmental scanning; cross-unit communication; risk signal detection; technology-enabled monitoring	Enhanced real-time monitoring; improved information-sharing; early-warning capability
Session 6: Adaptive capacity	Training; 6 h - longest session	Adaptive Capacity (18%) - second-highest weight	Scenario-based simulation exercises; resource reconfiguration drills; role flexibility training; contingency planning	Increased organizational flexibility; enhanced role redundancy; sustained core functions under disruption
Session 7: Learning processes	Training; 4 h	Learning Processes (13%)	After-action review protocols; organizational memory systems; knowledge transfer mechanisms; post-incident debriefing	Institutionalized learning cycles; improved knowledge retention; lessons integrated into operational protocols
Session 8: Organizational performance	Training; 3 h	Organizational Performance (11%)	OR performance indicators; ORQ-based benchmarking; KPI design; continuous improvement cycle embedding	Resilience KPI infrastructure; institutionalized tracking; capacity for ongoing self-assessment
**Phase II: Organizational embedding & change consolidation — 40 h over 24 weeks (8 sessions**,** one every 3 weeks; in-person or via Zoom)**				
Embedding & micro-interventions	Implementation; 40 h (total program = 70 h)	1–2 priority domains per unit; all domains at organizational level	Unit-level gap analysis; domain-specific micro-interventions; reflection meetings; cross-unit coordination	Sustainable OR change; embedded domain-specific routines; measurable OR improvement at T2 and T3

OR = organizational resilience; ORQ = Organizational Resilience Questionnaire; KPI = key performance indicator. ORQ-validated domain weights in parentheses. Total program: 70 h over approximately 7–8 months. T2 = post-intervention (May–June 2025); T3 = six-month follow-up (January–February 2026). EMS = emergency medical services

### Ethics

The study was approved by the Tel Aviv University Institutional Review Board (approval no. 0004464-7; 26 March 2024). Electronic informed consent was obtained from all participants prior to completing the survey. Data were collected anonymously and analyzed in aggregate.

### Statistical analysis

All analyses were conducted in IBM SPSS Statistics (version 29). ORQ internal consistency was assessed using Cronbach’s α at each time point. Between-time-point OR comparisons were conducted using independent-samples t-tests with Bonferroni correction applied across seven simultaneous comparisons (seven OR domains), yielding a corrected significance threshold of α* = 0.005 (99.5% CIs reported; Table 5). For the three independent organizational predictor variables, Bonferroni correction was applied separately across three simultaneous comparisons, yielding a corrected threshold of α* ≈ 0.017 (98.3% CIs reported; Table 6). Four sequential multivariable regression models were constructed on pooled data (*n* = 573) with dummy-coded year indicators (T2 = short-run; T3 = long-run; T1 = reference) and progressive entry of covariates, to assess the independent contribution of each covariate block to explained variance. An ANCOVA with Time (T1, T2, T3) and Profession (Paramedic vs. other) as between-group factors, and transformational leadership, employee well-being, technological advancement, and age as covariates, was specified to assess the independent intervention effect after full covariate adjustment (partial η² reported; Bonferroni-adjusted post-hoc comparisons). Statistical significance was set at *p* < 0.05 unless otherwise specified.

To assess multicollinearity among the three structural predictors, Variance Inflation Factors (VIF) were computed for each predictor in the final parsimonious model (Model 4). All VIF values were below 4.42 (range: 1.04–4.41), indicating no multicollinearity concern (conventional threshold: VIF < 10 [26]).

Transformational leadership, employee well-being, and technological advancement were incorporated into the measurement instrument as structural predictors prior to T1 data collection; the ORQ itself was developed and validated at T0 (2022) without these three constructs as subscales. In the present analysis, these variables are treated as pre-existing structural predictors of OR rather than as post-treatment mediators or confounders — a conceptualization empirically supported by minimal, statistically non-significant between-cohort differences across the study period (η²≤0.014; Table 4), indicating relative stability of these constructs over time. Their inclusion as covariates in the ANCOVA model is intended to improve the precision of the time-effect estimate. A partial mediating pathway cannot be definitively excluded.

## Results

### Sample characteristics and instrument reliability

Data were collected at three measurement time points within the EMS organization. T1 (January–February 2024; *n* = 196): pre-intervention baseline, serving as the primary reference point. T2 (May–June 2025; *n* = 205): primary post-intervention assessment, administered upon completion of the 70-hour intervention program. T3 (January–February 2026; *n* = 172): six-month post-program follow-up, assessing the durability of intervention effects.

Table 2 presents Cronbach’s alpha values for the composite ORQ and its seven domains across the three measurement time points. Composite reliability was excellent across all time points (α = 0.965–0.969), and domain-level reliability ranged from α = 0.648 to α = 0.916.

**Table 2:** Internal consistency reliability of the organizational resilience questionnaire (ORQ) and its domains across three measurement time points

ORQ domain	No. of items	T1	T2	T3
**Composite ORQ - all domains**	29	0.965 (0.966)	0.967 (0.968)	0.969 (0.970)
Ethos	3	0.711 (0.718)	0.648 (0.667)	0.704 (0.719)
Organizational culture	4	0.821 (0.821)	0.832 (0.832)	0.847 (0.851)
Leadership and human capital	2	0.689 (0.701)	0.662 (0.676)	0.701 (0.707)
Situational awareness	7	0.882 (0.887)	0.858 (0.864)	0.889 (0.894)
Adaptive capacity	7	0.899 (0.902)	0.915 (0.921)	0.916 (0.920)
Organizational performance	3	0.843 (0.844)	0.856 (0.857)	0.831 (0.831)
Learning processes	3	0.833 (0.833)	0.832 (0.832)	0.836 (0.840)

Values represent Cronbach’s alpha. Values in parentheses represent Cronbach’s alpha based on standardized items. T1 = pre-intervention baseline; T2 = post-intervention assessment; T3 = six-month follow-up. ORQ = Organizational Resilience Questionnaire

Prior to the main analyses, the comparability of the three cohorts was examined to ensure that the observed OR changes were not attributable to compositional differences between samples (Table 3).

**Table 3:** Sample characteristics and between-cohort comparisons across three time points within the EMS organization

		T1–2024 (*n* = 196)	T2–2025 (*n* = 205)	T3–2026 (*n* = 172)		Cramér’s V
Categorical & ordinal variables: n (%)					P value	
Age	Up to 30	77 (39.3)	111 (54.1)	64 (37.2)	0.001	0.154
	Over 30	119 (60.7)	94 (45.9)	108 (62.8)		
Gender	Women	52 (26.5)	82 (40.2)	55 (32)	0.014	0.120
	Men	144 (73.5)	122 (59.8)	117 (68)		
Ethnicity	Jews	191 (97.4)	198 (96.6)	167 (97.1)	0.87	0.022
	Others	5 (2.6)	7 (3.4)	5 (2.9)		
Education	Academic	86 (43.9)	72 (35.1)	75 (43.6)	0.13	0.084
	Non-academic	110 (56.1)	133 (64.9)	97 (56.4)		
Income level	Below average	90 (45.9)	79 (38.5)	65 (37.8)	0.043	0.093
	Average	72 (36.7)	75 (36.6)	54 (31.4)		
	Above average	34 (17.3)	51 (24.9)	53 (30.8)		
Religion level	Secular	68 (35.2)	71 (34.6)	62 (36)	0.16	0.082
	Traditional	62 (32.1)	63 (30.7)	50 (29.1)		
	Religious	63 (32.6)	65 (31.7)	53 (30.8)		
	Other	0	6 (2.9)	7 (4.1)		
Role	Managerial	12 (6.1)	8 (3.9)	5 (2.9)	0.33	0.065
	Non-managerial	184 (93.9)	197 (96.1)	167 (97.1)		
Seniority	Up to 1 year	15 (7.7)	48 (23.4)	27 (15.7)	< 0.001	0.155
	1–5 years	73 (37.2)	82 (40)	55 (32)		
	6–10 years	48 (24.5)	40 (19.5)	46 (26.7)		
	More than 10	60 (30.6)	35 (17.1)	44 (25.6)		

p values from chi-square tests of independence across the three cohorts. Effect sizes: Cramér’s V. EMS = emergency medical services

### Composite organizational resilience across three time points

The mean composite OR scores were: at baseline (T1) 3.86 ± 0.73, at post-intervention (T2) 4.06 ± 0.70, and at six-month follow-up (T3) 4.09 ± 0.63. A one-way ANOVA revealed a significant overall difference across the three measurement occasions (F(2, 570) = 5.72, *p* = 0.004). Bonferroni-corrected pairwise comparisons indicated a significant short-term increase in OR scores from T1 to T2 ( Δ = +0.20, *p* = 0.018, d = 0.27), consistent with a potential intervention-related improvement, although causal attribution cannot be established from the present design, and a significant and numerically larger long-term increase from T1 to T3 (Δ = +0.23, *p* = 0.007, d = 0.32). The T2-to-T3 comparison was non-significant (Δ = +0.03, *p* = 0.990).

As shown in Table 4, transformational leadership differed significantly across cohorts (*p* = 0.015), while employee well-being and technological advancement showed no significant between-cohort differences (*p* = 0.230 and *p* = 0.530, respectively); effect sizes were small across all three variables (η² ≤ 0.014).

**Table 4:** Descriptive statistics and between-cohort comparisons for the three continuous independent variables across measurement time points — EMS organization

Independent variable	T1 — 2024 (*n* = 196) Mean (SD)	T2 — 2025 (*n* = 205) Mean (SD)	T3 — 2026 (*n* = 172) Mean (SD)	*p*	η²
Transformational leadership	3.53 (0.84)	3.74 (0.88)	3.76 (0.90)	0.015	0.014
Employee well-being	3.95 (0.74)	4.05 (0.76)	4.08 (0.73)	0.230	0.005
Technological advancement	3.69 (0.85)	3.73 (0.96)	3.79 (0.91)	0.530	0.002

Values are means (standard deviations). p-values from one-way ANOVA. Effect sizes are reported as η². All variables are measured on a 1–5 scale. EMS = emergency medical services

### Domain-level organizational resilience across three time points: T1→T2 and T1→T3

**Fig. 1:**
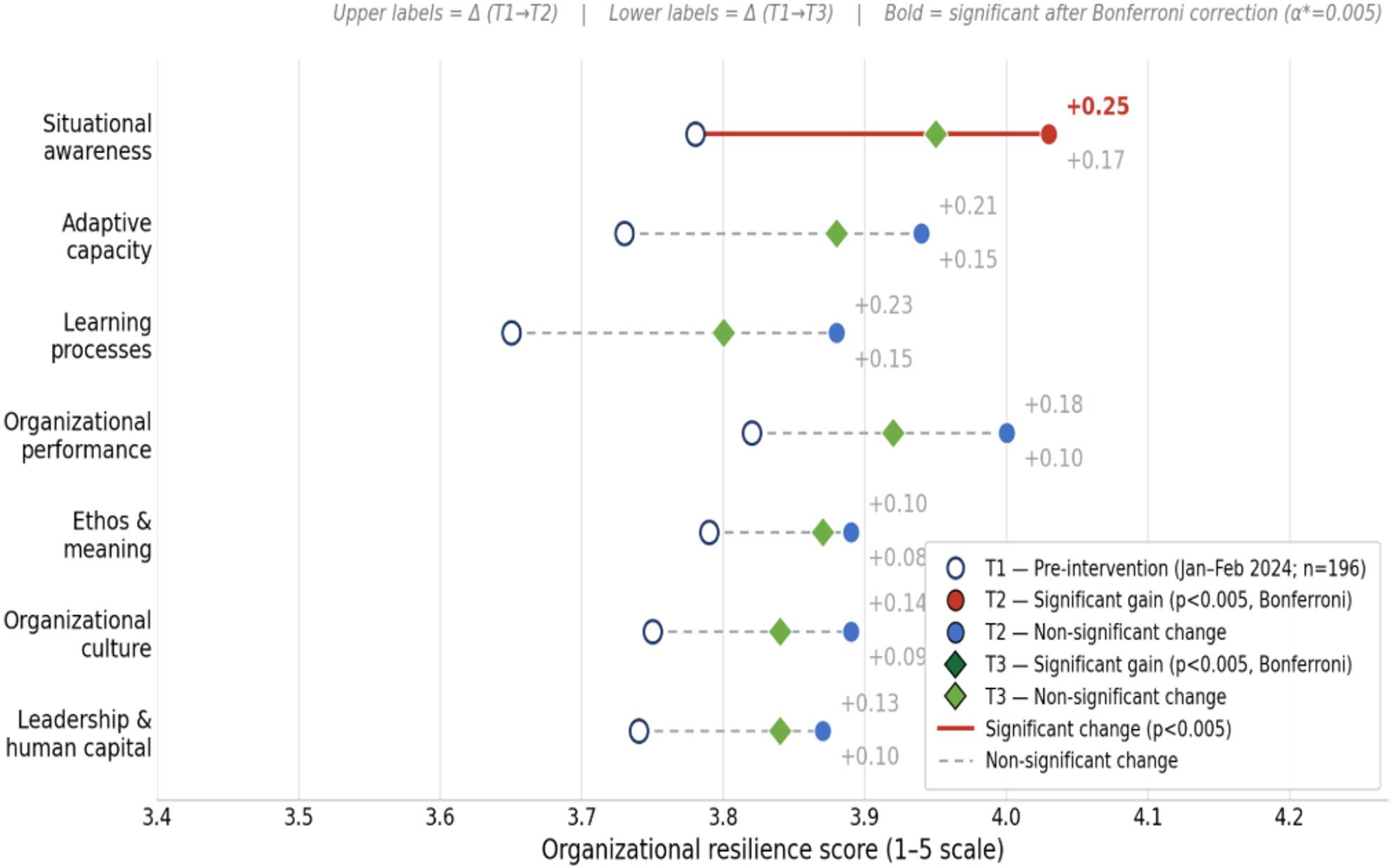
Domain-level organizational resilience scores across three measurement time points (T1, T2, and T3) for the seven OR domains within the EMS organization

Domain-level changes from T1 to T2 and from T1 to T3 (six-month follow-up) were examined using independent t-tests with Bonferroni correction applied across seven comparisons (significance threshold: α* = 0.005; 99.5% CIs reported). At T2, compared to T1, one domain - situational awareness - reached statistical significance after Bonferroni correction (Δ=+0.25, *p* < 0.001; α*=0.005). Two additional domains reached conventional but not Bonferroni-corrected significance [27]: adaptive capacity (Δ=+0.21, *p* = 0.006) and learning processes (Δ=+0.23, *p* = 0.006). Organizational performance reached significance without Bonferroni correction (*p* = 0.030). The remaining three domains showed positive but non-significant trends (*p* = 0.06–0.28). Table 5 presents the full domain-level results for the seven OR domains across both comparisons (T1→T2 and T1→T3). The pattern was internally coherent with the ORQ-weighted curriculum structure (Fig. 1), which displays all three measurement time points for the seven OR domains.

**Table 5:** Domain-level organizational resilience changes across measurement time points (T1→T2 and T1→T3) — EMS organization (Bonferroni-corrected, α*=0.005; 99.5% CI reported)

Domain	T1 → T2 (Post-intervention)				T1 → T3 (Six-month follow-up)			
	Δ	t	99.5% CI	*p*	Δ	t	99.5% CI	*p*
Situational awareness	+ 0.25	3.84	[0.08, 0.42]	**< 0.001**	+ 0.17	2.38	[− 0.02, 0.36]	0.009
Adaptive capacity	+ 0.21	2.56	[0.01, 0.44]	0.006	+ 0.15	1.70	[− 0.08, 0.38]	0.045
Learning processes	+ 0.23	2.54	[0.01, 0.48]	0.006	+ 0.15	1.55	[− 0.11, 0.42]	0.016
Organizational performance	+ 0.18	1.89	[− 0.07, 0.43]	0.030	+ 0.10	1.03	[− 0.16, 0.36]	0.150
Ethos and meaning	+ 0.10	1.55	[− 0.07, 0.28]	0.060	+ 0.08	1.10	[− 0.11, 0.28]	0.130
Organizational culture	+ 0.14	1.57	[− 0.09, 0.38]	0.060	+ 0.09	1.01	[− 0.15, 0.34]	0.150
Leadership and human capital	+ 0.13	1.48	[− 0.11, 0.38]	0.070	+ 0.10	0.97	[− 0.17, 0.37]	0.160

Domains sorted by T1→T2 t-statistic magnitude. Bold p-values indicate significance after Bonferroni correction (α*=0.005). Δ = mean difference relative to T1 (pre-intervention baseline). OR = organizational resilience; EMS = emergency medical services

Open circles (white) represent T1 (pre-intervention baseline; *n* = 196); filled circles represent T2 (post-intervention; *n* = 205); diamonds represent T3 (six-month follow-up; *n* = 172). Solid connecting lines indicate statistically significant change (*p* < 0.005, Bonferroni-corrected); dashed lines indicate non-significant change. Upper Δ labels = T1→T2; lower Δ labels = T1→T3. OR = organizational resilience; EMS = emergency medical services.

Domain-level changes were also examined for the three continuous independent variables theorized to predict OR — transformational leadership, employee well-being, and technological advancement — across T1→T2 and T1→T3, using the same independent samples one-way analysis of variance (ANOVA) procedure with Bonferroni correction (α* = 0.017; 98.3% CIs reported). Transformational leadership showed significant gains at both T2 (Δ=+0.21, *p* = 0.006) and T3 (Δ=+0.23, *p* = 0.005). Employee well-being and technological advancement demonstrated positive but non-significant trends at both time points. Table 6 presents the full results.

**Table 6:** Domain-level changes across measurement time points (T1→T2 and T1→T3) for the three independent variables — EMS organization (Bonferroni-corrected, α*=0.017; 98.3% CI reported)

	Change from T1 to T2				Change from T1 to T3			
	Delta	t	98.3% C.I	*p*.v	Delta	t	98.3% C.I	*p*.v
Transformational leadership	+ 0.21	2.51	[0.01, 0.41]	**0.006**	+ 0.23	2.59	[0.07, 0.45]	**0.005**
Technological advancement	+ 0.05	0.56	[-0.16, 0.27]	0.28	+ 0.11	1.15	[-0.11, 0.33]	0.12
Employee well-being	+ 0.1	1.32	[-0.08, 0.28]	0.09	+ 0.12	1.63	[-0.06, 0.31]	0.06

Δ = mean difference relative to T1 (pre-intervention baseline). Bold p-values indicate significance after Bonferroni correction (α*≈0.017). OR = organizational resilience; EMS = emergency medical services

### Identifying predictors of organizational resilience over time

To identify the independent predictors of OR and examine the robustness of the intervention effect after covariate adjustment, four sequential multivariable linear regression models were constructed using data pooled across all three time points (*n* = 573 observations; Year coded as dummy variables: 2025 [short-run] and 2026 [long-run] relative to 2024 as reference). Model 1 included only the time indicators; Model 2 added sociodemographic covariates; Model 3 added the three key organizational predictors (transformational leadership, employee well-being, technological advancement); Model 4 retained only the variables significant at α ≤ 0.05 in the full model. Table 7 presents standardized β coefficients and p-values for all four models. Bold p-values indicate a significance corrected for multiple comparisons.

**Table 7:** Sequential multivariable regression models predicting organizational resilience — EMS organization (T1, T2, T3 pooled; *n* = 573)

	Model #1		Model #2		Model #3		Model #4	
	Beta	*p*.v	Beta	*p*.v	Beta	*p*.v	Beta	*p*.v
Year: 2025 [short run]	0.136	**0.004**	0.104	**0.026**	0.06	**0.014**	0.06	**0.007**
Year: 2026 [long run]	0.147	**0.002**	0.135	**0.004**	0.057	**0.014**	0.057	**0.013**
Gender: male	---	---	-0.117	**0.006**	-0.0122	0.289	---	---
Ethnicity: Jew	---	---	-0.017	0.675	-0.019	0.345	---	---
Academic education	---	---	-0.178	**< 0.001**	-0.014	0.54	---	---
Age (years)	---	---	0.074	0.107	0.069	**0.003**	0.038	**0.004**
Profession: Medic	---	---	-0.162	**< 0.001**	-0.029	0.148	---	---
Transformational leadership	---	---	---	---	0.441	**< 0.001**	0.458	**< 0.001**
Technological advancement	---	---	---	---	0.222	**< 0.001**	0.208	**< 0.001**
Employee well-being	---	---	---	---	0.271	**< 0.001**	0.274	**< 0.001**
**F statistical**	F(2, 569) = 5.98; *p* < 0.001		F(7, 564) = 8.7; *p* < 0.001		F(10, 561) = 192.49; *p* < 0.001		F(6, 566) = 319.5; *p* < 0.001	
**R²**	**R²= 0.021**		**R²= 0.097**		**R²= 0.77**		**R²= 0.77**	

β = standardized regression coefficient. Reference category for Year: T1 (2024). — = variable not included in model. Additional unstandardized coefficients, 99.1% confidence intervals, and VIF values for the final parsimonious model are presented in Table 7 (supplement). Bold = statistically significant (*p* < 0.05). Year: 2025 = short-run intervention effect; Year: 2026 = long-run intervention effect. EMS = emergency medical services

A consistent pattern across the sequential models was that both time indicators - Year: 2025 (short-run) and Year: 2026 (long-run) - remained statistically significant at *p* ≤ 0.05 in every model specification, from the unadjusted Model 1 through the fully adjusted Models 2, 3, and 4. This consistency across model specifications suggests that the observed improvement in OR over time was not fully accounted for by the sociodemographic or organizational covariates included in the analysis. In the final parsimonious model (Model 4), the three organizational predictors and age together accounted for the bulk of explained variance (β: transformational leadership = 0.458, employee well-being = 0.274, technological advancement = 0.208, age = 0.038; all *p* ≤ 0.050), with the time indicators retaining significance at *p* < 0.05 (β(2025) = 0.060, *p* = 0.007; β(2026) = 0.057, *p* = 0.013). The full model explained 77.0% of the OR variance (R² = 0.770).

### Intervention effects controlling for organizational and individual covariates

To assess the independent effect of the intervention program controlling for all key covariates, an Analysis of Covariance (ANCOVA) was conducted with Time (T1, T2, T3) and Profession (Paramedic vs. other) as between-group factors, and transformational leadership, employee well-being, technological advancement, and age as covariates (R²=0.772). Table 8 presents the full ANCOVA output.

**Table 8:** ANCOVA predicting organizational resilience across measurement time points (T1, T2, T3) — EMS organization

Source	d.f	F	*p* value	ηᵖ²
Intercept	(1, 563)	108.58	< 0.001	0.162
Transformational leadership (covariate)	(1, 563)	104.16	< 0.001	0.156
Employee well-being (covariate)	(1, 563)	67.18	< 0.001	0.107
Technological advancement (covariate)	(1, 563)	38.50	< 0.001	0.064
Age in years (covariate)	(1, 563)	8.13	0.005	0.014
Time (T1, T2, T3)	(2, 563)	7.41	0.001	0.026
Profession: Medic (vs. other)	(1, 563)	1.61	0.205	0.003
Time × Profession: Medic	(2, 563)	3.09	0.046	0.011
Error	563			
Total	573			
R² = 0.772				

ηᵖ² = partial eta-squared; df = degrees of freedom, reported as (df_effect, df_error). Covariates (transformational leadership, employee well-being, technological advancement, and age) were entered simultaneously; pairwise comparisons were Bonferroni-adjusted. Error df = 563 for all sources. EMS = emergency medical services

#### Time effect: short- and long-term intervention gains

Even after controlling for all covariates, the Time factor remained a significant independent predictor of OR (F(2, 563) = 7.41, *p* = 0.001, ηᵖ²=0.026). Bonferroni-adjusted pairwise comparisons revealed significant covariate-adjusted gains at both T2 (3.86 vs. 4.06, *p* = 0.008) and T3 (3.86 vs. 4.09, *p* = 0.002) relative to the pre-intervention baseline. The T2-to-T3 comparison was non-significant (*p* = 0.990).

#### Profession effect and time × profession interaction

With respect to the professional role, the main effect of Profession did not reach significance after covariate adjustment (F = 1.61, *p* = 0.205, ηᵖ²=0.003). Unadjusted means indicate that paramedics (overall mean = 3.96) entered with a somewhat lower baseline OR than other staff and volunteers (overall mean = 4.01), a gap that was substantially reduced by T3.

The Time × Profession interaction was significant (F = 3.09, *p* = 0.046, ηᵖ²=0.011), indicating that OR trajectories differed between professional groups across measurement occasions. Paramedics entered with the lowest adjusted baseline OR (3.80), showed a substantial gain at T2 (4.01), and continued to improve at T3 (4.07), yielding a net T1→T3 gain of + 0.27. Other staff and volunteers entered with a higher baseline OR (3.97), increased modestly at T2 (4.04), and showed a marginal decline at T3 (4.02), yielding a net gain of + 0.05.

**Fig. 2:**
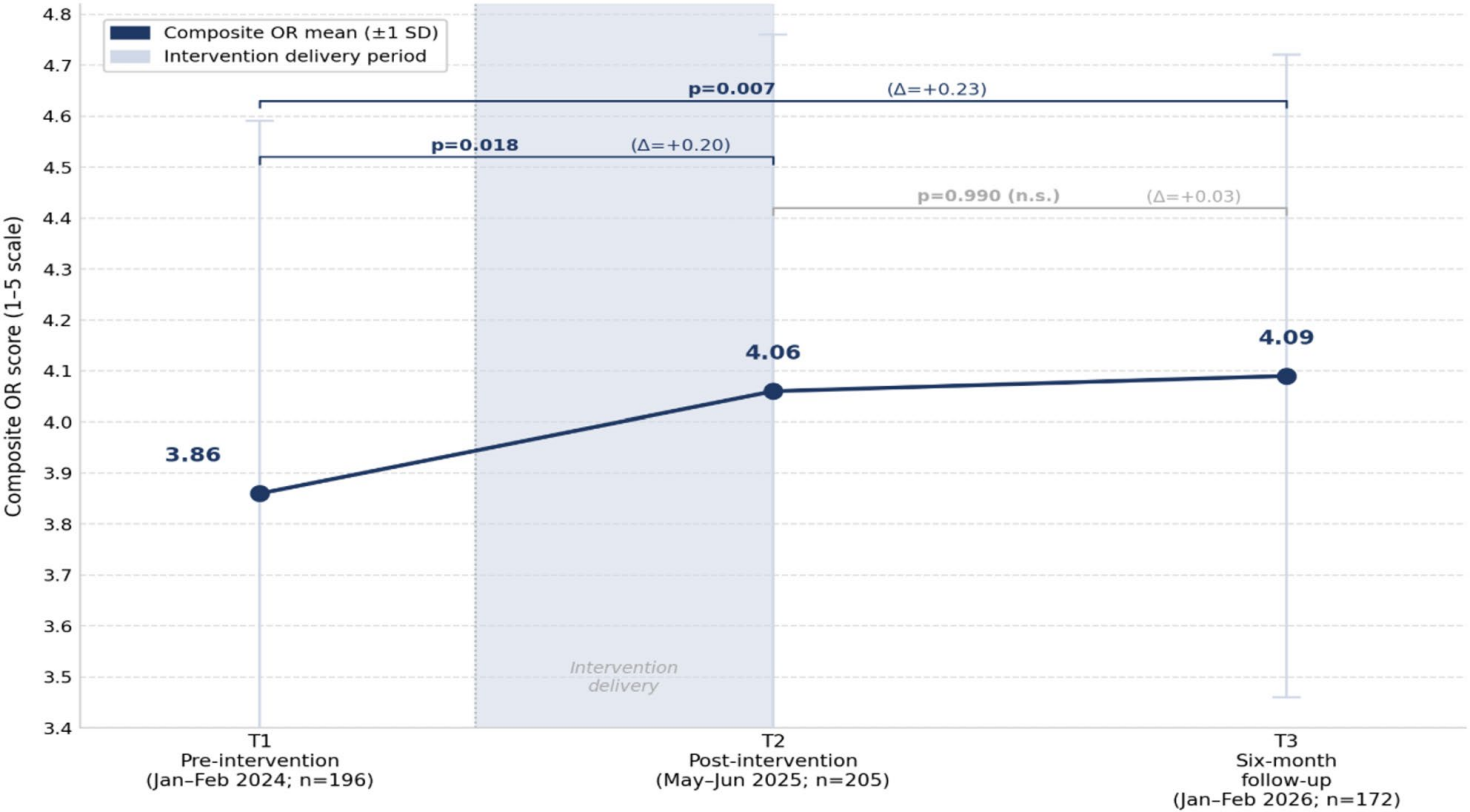
Composite organizational resilience scores across three measurement time points within the EMS organization. T1 = pre-intervention baseline (*n* = 196); T2 = post-intervention assessment (*n* = 205); T3 = six-month follow-up (*n* = 172). Error bars represent ± 1 standard deviation. OR = organizational resilience; EMS = emergency medical services

Figure 2 presents the longitudinal trajectory of the composite OR score across the three study measurements — from the pre-intervention baseline (T1) through the six-month follow-up (T3), presenting a direct visual comparison of effect magnitude and durability across the full intervention period.

## Discussion

### Principal findings

This study examined organizational resilience changes associated with the implementation of a 70-hour OR intervention within an Israeli EMS organization operating under protracted conflict conditions. All three pre-specified hypotheses were confirmed: OR improved significantly following the intervention; those gains were maintained and further consolidated at six-month follow-up; and transformational leadership, employee well-being, and technological advancement emerged as the dominant organizational predictors of OR across all measurement occasions. Notably, progressive consolidation of OR gains from T1 to T3 represents the most substantively important trajectory in this study: the T1→T3 gain was larger in magnitude than the T1→T2 gain, suggesting that the Phase II embedding component extended and deepened the intervention’s impact beyond the formal training endpoint. Paramedics - the professional subgroup with the highest operational exposure and the lowest baseline OR - demonstrated the largest T1→T3 gains of all professional groups, a finding that underscores the intervention’s relevance for frontline emergency professionals confronting the greatest organizational stressors [28, 29].

### Intervention effectiveness and domain-level coherence

The post-intervention gains were achieved under continuous conflict-related operational stress. Two interpretations warrant acknowledgement: first, conflict conditions may have suppressed OR improvement by amplifying operational demands and burnout, leading to underestimation of the intervention’s potential in lower-stress contexts. Second, the heightened salience of organizational security under active conflict may have independently elevated self-reported resilience awareness, potentially inflating the magnitude of observed OR gains independently of training content. Without a concurrent comparison group under equivalent conflict exposure, these competing hypotheses cannot be distinguished [1, 2, 3].

The pattern of domain-level gains was internally coherent with the ORQ-weighted curriculum: Significant gains at T2 were observed specifically in the domains receiving the most intensive programmatic attention - situational awareness, adaptive capacity, and learning processes - all of which are among the highest-weighted domains in the ORQ curriculum. This domain-specific pattern of improvement is unlikely to be explained simply by participants’ awareness that they were being observed and evaluated; rather, it suggests that the curriculum content itself may have served as an active driver of change [30]. The non-significant gain in technological advancement is consistent with the possibility that technology-related OR improvement may depend less on training alone and more on sustained structural infrastructure investment, though this interpretation requires caution given the single-domain null finding [31, 32].

### Six-month follow-up and differential professional trajectories

The six-month follow-up demonstrated that OR gains were not only maintained but further strengthened relative to baseline, consistent with a pattern of progressive consolidation: the Phase II embedding component appears to have contributed to sustained OR gains beyond the formal training endpoint [13, 16, 33]. The significant Time × Profession interaction (ηᵖ²=0.011, *p* = 0.046) should be interpreted with caution, given its small effect size and the constraints of the cross-sectional design. Higher OR scores were observed among paramedic cohorts at T2 and T3 relative to T1, a trend not observed to the same extent among other professional groups. This association may reflect differential indirect exposure to trustee-led activities, profession-specific socialization during the conflict period, or unmeasured operational confounds [28, 29, 34]. Several features of the intervention plausibly account for the stronger response among paramedics. As the professional group with the lowest adjusted baseline OR and the highest operational and trauma exposure, they had the greatest scope for measurable improvement; moreover, the most intensively emphasized program components — situational awareness, adaptive capacity (scenario-based simulation), and learning processes (after-action reviews and post-incident debriefing) — map directly onto the field-level realities they confront daily. As the cohort that effectively leads and manages operations in the field, paramedics were also positioned both to exercise and to benefit from the program’s transformational-leadership content (personal example, openness, and structured dialogue), which was the single dominant predictor of OR in this study (β = 0.458). These elements may have rendered the intervention more immediately salient to frontline crews than to staff in non-operational roles. Future iterations should tailor content intensity to professional role subgroups.

### Structural predictors

Transformational leadership, employee well-being, and technological advancement were consistently the dominant OR predictors across the pooled dataset spanning all three measurement occasions, robust to full covariate adjustment. These findings align with evidence on the central role of transformational leadership in fostering healthcare resilience and adaptive capacity, employee well-being as a predictor of sustained organizational functioning under stress, and technological advancement as an enabling infrastructure for organizational responsiveness [35, 36, 37]. Notably, the time indicators retained significance in every model, suggesting that the observed OR change over time was not fully accounted for by these structural predictors. Rather, the significant and consistent time effect across all model specifications suggests that the intervention itself contributed to OR improvement beyond what could be explained by pre-existing organizational and demographic characteristics.

### Situating findings within the broader evidence base

Systematic reviews of OR measurement have consistently identified a lack of controlled intervention studies at the organizational level [12, 18], and the most recent scoping review found no published longitudinal interventional studies specifically targeting pre-hospital EMS [13]. The present study was designed to address that gap. In contrast to the web-based multi-level delivery model reported by Gil-Hernández et al.^17^ and the public-sector training frameworks synthesized by Hollaar et al.,^16^ the present study grounded its curriculum priorities in an empirically validated, domain-weighted ORQ hierarchy — a more targeted, domain-specific approach relative to generic resilience programs. A further distinctive feature lies in its deployment of internal Resilience Trustees as embedded change agents [25]. Implementation science emphasizes that internal change agents - individuals embedded within the organization who carry authority, credibility, and sustained commitment - are more effective at institutionalizing complex innovations than externally delivered programs. In the present study, the Resilience Trustees served precisely this function: trained internal role-holders who continued to drive OR-building across stations and units beyond the formal intervention period, without dependence on external facilitation. This design choice may help explain the progressive consolidation of OR gains at T3 and suggests that the Trustee model could represent a promising, self-sustaining mechanism for OR institutionalization in healthcare organizations [25, 38].

### Cohort comparability and covariate adjustment

As is common in quasi-longitudinal organizational research with voluntary participation, some between-cohort demographic variation was observed across measurement occasions. The analytical design addressed this directly: sequential multivariable regression and ANCOVA models incorporated transformational leadership, employee well-being, technological advancement, and age as covariates, thereby statistically controlling for compositional differences across cohorts. The time effect remained significant across all model specifications, including the fully adjusted ANCOVA, providing evidence that the observed OR trajectory reflected substantive organizational change and was not merely a function of demographic variation between samples [39].

The elevated representation of newer, shorter-tenure staff at T2 constitutes a demographic compositional difference consistent with documented associations between organizational tenure, socialization, and OR levels [28, 29]; however, individual-level response-pattern data by staff cohort were not collected, and the precise mechanism underlying this compositional shift cannot be established with certainty. While age and seniority were entered as ANCOVA covariates to mitigate this effect, residual compositional influence on between-cohort comparisons is acknowledged as a limitation.

### Policy implications

Three policy considerations follow from the findings. First, OR investment may warrant consideration as a continuous strategic priority: the progressive consolidation trajectory provides empirical support for sustained, pre-emptive OR investment rather than solely reactive post-crisis programming [16, 33]. Second, transformational leadership may warrant consideration as a candidate national-level investment priority within EMS management pathways and, more broadly, across healthcare organizations engaged in OR planning. With the highest standardized coefficient among all modifiable predictors (β = 0.458, *p* < 0.001) and consistent dominance across all three measurement occasions, it represents the most powerful organizational lever for OR improvement identified in this study. The findings support exploring its formal integration as a candidate Key Performance Indicator within national resilience monitoring frameworks, pending replication in controlled, multi-organization studies; validated instruments — such as the LQ Model used in the present study — could be considered for periodic assessment within the Ministry of Health infrastructure. The Resilience Trustee model offers a directly implementable vehicle for distributing transformational leadership competencies at the team level, without requiring executive-level succession planning [35, 36, 37]. Third, employee well-being and technological advancement may warrant recognition as OR-relevant organizational priorities, supporting the need for sustained attention to peer-support governance, psychological safety practices, and operational infrastructure [31, 32, 40]. From an organizational governance perspective, the present findings support two concrete policy instruments. Healthcare organizations — particularly those operating under chronic operational stress, may benefit from adopting the ORQ as a routine internal monitoring tool: administered periodically, it provides a structured, validated mechanism for tracking OR levels across seven organizational domains, identifying vulnerabilities, and informing resource allocation decisions. Additionally, the Resilience Trustee-based intervention program, supported by preliminary effectiveness evidence, provides a directly implementable, organizationally embedded model for OR strengthening that does not require sustained external expertise. Once a cohort of internal trustees is trained, the program can be delivered and sustained internally across stations and units. Healthcare policymakers are encouraged to consider embedding such structured OR development programs within regular workforce development cycles, and to evaluate the ORQ as a population-level indicator of organizational health in conflict-affected and high-stress healthcare settings [19, 20]. However, further research using controlled and multi-site designs is needed before recommending widespread adoption.

Program feasibility during routine operations warrants brief comment. The 70-hour commitment was delivered in two structurally distinct parts — a concentrated training block (Phase I; 30 h) comprising four full-day sessions, and an extended embedding and change-accompaniment phase (Phase II; 40 h) distributed across approximately six months as eight shorter sessions held once every three weeks (in person or via Zoom). Critically, this commitment applied only to the designated cadre of 20 Resilience Trustees within an intervention district of approximately 1,100 employees of varied roles — roughly 2% of the workforce — so that the broader field staff were not withdrawn from operational duty for extended training. To preserve continuity of function and a constant state of high readiness, deputies were formally appointed to cover the trustees’ operational roles during the concentrated Phase I block. Agencies seeking to scale the model may further stagger trustee scheduling across stations and shifts, integrate sessions into existing protected-time or continuing-education allocations, and use remote delivery for Phase II. Because the model becomes self-sustaining once an internal trustee cohort is trained, the 70-hour investment is incurred once per cohort rather than recurrently.

### Strengths and limitations

This study has several notable strengths: a three-time-point longitudinal design with repeated measurement occasions spanning 24 months; use of a validated, domain-weighted instrument (ORQ) with demonstrated internal consistency across all three cohorts; deployment of both multivariable regression and ANCOVA to control for organizational and sociodemographic confounders; and the ecologically valid conflict-affected setting, which provides a stringent real-world test of OR intervention effects.

Nonetheless, several limitations should be noted. The present study constitutes an initial effectiveness study providing preliminary evidence of OR improvement associated with the Resilience Trustee program. Design limitations include: no randomization at any level; implementation within a single EMS organization; and a repeated cross-sectional design, discussed further below. Additionally, self-reported data are subject to social desirability bias and common-method variance. The high proportion of OR variance explained by the three structural predictors (R²=0.770–0.772) may partially reflect common-method variance, given that all predictor and outcome measures were drawn from the same self-report instrument. Future studies should supplement ORQ scores with objective organizational performance indicators — such as incident response times or protocol adherence rates — to reduce reliance on single-source data [41, 42]. To guide future implementation, such indicators may be organized around the three independent predictors of OR identified here: for transformational leadership, the coverage and frequency of structured after-action reviews and post-incident debriefs led by shift commanders, documented developmental and feedback conversations per supervisor, and periodic leadership-behavior assessment; for technological advancement, adoption and utilization rates of digital operational and clinical systems, system reliability, and time-to-integration of new protocols; and for employee well-being, burnout-related absenteeism and sick-leave rates, staff turnover and retention, and uptake of peer-support and psychological-safety mechanisms. These predictor-aligned indicators, tracked alongside operational and clinical performance measures such as unit response and turnaround intervals and clinical-protocol adherence, would allow objective signals to corroborate self-reported OR gains. The temporal overlap of all measurement occasions with the ongoing conflict is a fundamental epistemic constraint. The study employed a repeated cross-sectional design rather than a true panel design, meaning that different individuals responded at each time point; while this is a known limitation of quasi-longitudinal organizational research, it is particularly relevant to this study, as the ORQ measures organizational-level resilience rather than individual resilience, and prior validation work supports the use of cross-sectional aggregates for this purpose [20]. The single-organization design limits generalizability, and the contextual specificity of the Israeli conflict environment may constrain direct transferability.

Fundamentally, the repeated cross-sectional design precludes causal inference. While OR scores increased significantly from T1 to T3 concurrent with the intervention, these associations may reflect: (a) compositional change, whereby the T2 cohort’s relative youth may have independently elevated self-reported OR; (b) conflict-context adaptation, whereby natural organizational adjustment to prolonged stress may have elevated OR levels independently of the intervention; or (c) Hawthorne effects, whereby repeated measurement may have heightened staff awareness of resilience practices regardless of program participation. Findings should accordingly be interpreted as associations between the intervention and OR improvement, not as evidence of causal effects.

All three measurement occasions overlapped with the ongoing armed conflict, and conflict dynamics varied across the study period, with periods of escalation and relative stabilization between T1 (January 2024), T2 (May–June 2025), and T3 (January 2026). These fluctuations may have introduced systematic variation in OR self-reports unrelated to the intervention. The independent contribution of conflict dynamics to the observed OR trajectory cannot be isolated in the absence of a concurrent non-intervention comparison group.

Additionally, between-cohort demographic variation was observed across measurement occasions; notably, the T2 cohort was markedly younger and of shorter organizational tenure than the T1 and T3 cohorts — a pattern consistent with documented associations between organizational tenure, socialization, and OR levels, as discussed in the Cohort Comparability subsection. Individual-level data were not collected, however, and the precise mechanism underlying this compositional shift cannot be established with certainty. While ANCOVA models adjusted for age, the residual effect of compositional differences on OR scores cannot be fully excluded. Time-point comparisons should accordingly be interpreted as organizational-level associative patterns, not as evidence of individual-level longitudinal change. Sensitivity analyses stratified by age or seniority would be informative in future work [28, 29].

A related limitation concerns the granularity of exposure data. Although all respondents were members of the intervention district and were exposed to the organizational resilience strengthening project at the district level through awareness activities and selective program participation, the precise degree of individual engagement — whether full, partial, or minimal — was not systematically tracked. Relatedly, the residual inter-unit variation in implementation depth that is inherent to any distributed change-agent model was not formally quantified. Future research designs should incorporate individual-level exposure assessments alongside organizational-level outcome measurement to enable more precise attribution of observed OR changes to specific program components.

Future studies should incorporate mixed-methods process evaluation, objective operational performance indicators, and multi-organization replication [43, 44].

## Conclusions

Organizational resilience appears to be a measurable, modifiable, and structurally influenced capacity of EMS confronting chronic operational disruption. The present study examined OR changes associated with a designated intervention program implemented in a pre-hospital EMS operating under protracted conflict conditions, addressing a recognized gap in longitudinal interventional evidence in this setting.

The structured OR program produced statistically significant and domain-coherent resilience gains immediately following implementation, with those gains not only maintained but progressively consolidated at six-month follow-up.

Transformational leadership, employee well-being, and technological advancement were consistently and independently associated with OR across all three measurement occasions, indicating these as structural correlates that warrant dedicated institutional attention. These findings suggest that transformational leadership competencies may warrant formal embedding within EMS management development pathways; that employee well-being warrants recognition as a structural precondition for organizational resilience; and that technological advancement may require sustained infrastructure investment extending beyond the scope of any training program alone.

The ORQ and the Resilience Trustee intervention model — for which the present study provides preliminary effectiveness evidence — together offer a promising framework for OR strengthening in EMS and broader healthcare organizations confronting chronic operational disruption. Establishing a more robust evidence base would benefit from controlled or comparative designs, multi-organization replication across diverse healthcare settings, and integration of objective operational performance indicators alongside validated self-report instruments. In addition, because the structural predictors cannot be fully excluded as partial mediators of intervention-associated OR change, formal mediation analysis is recommended in future research to clarify these pathways.

## Supplementary Information

Below is the link to the electronic supplementary material.

Supplementary Material 1.

## Data Availability

The datasets generated and analysed during the current study are not publicly available, as the data were collected under conditions of participant anonymity and organizational confidentiality within an operational emergency medical service, in accordance with the terms of ethical approval granted by the Tel Aviv University Institutional Review Board (no. 0004464-7). De-identified data may be made available from the corresponding author upon reasonable request and subject to approval by the relevant institutional authority.

## Abbreviations

EMS: Emergency medical services
NIHP: Israel National Institute for Health Policy Research
IJHPR: Israel Journal of Health Policy Research
OR: Organizational resilience
ORQ: Organizational Resilience Questionnaire
T1: Pre-intervention baseline measurement (January–February 2024)
T2: Post-intervention measurement (May–June 2025)
T3: Six-month follow-up (January–February 2026)
