# Supplementary material for: Enhancing organizational resilience of emergency medical services through a designated intervention program
Source: Isr J Health Policy Res. 2026 Jul 23;15:29. doi: 10.1186/s13584-026-00772-1 (PMC13393947; doi:10.1186/s13584-026-00772-1)
Supplement: Supplementary file 1 — Supplementary Material 1. [file 13584_2026_772_MOESM1_ESM.docx]

**Table 7 (supplement).** Final parsimonious regression model predicting organizational resilience: unstandardized coefficients, confidence intervals, and collinearity diagnostics.

| **Predictor** | **B** | **SE** | **99.1% CI for B** | **VIF** |
| --- | --- | --- | --- | --- |
| Year: 2025 [short run] | .092 | .034 | [.003, .182] | 1.361 |
| Year: 2026 [long run] | .088 | .035 | [-.005, .181] | 1.336 |
| Age (years) | .003 | .001 | [.000, .005] | 1.045 |
| Transformational leadership | .367 | .034 | [.279, .455] | 4.412 |
| Employee well-being | .258 | .032 | [.175, .341] | 2.828 |
| Technological advancement | .161 | .026 | [.091, .230] | 2.933 |

*Note.* B = unstandardized regression coefficient; SE = standard error; CI = confidence interval for B; VIF = variance inflation factor. The table presents the final parsimonious regression model predicting organizational resilience using pooled data across T1, T2, and T3. The reference category for the year indicators is T1 (2024). The final model retained the short-run time indicator, the long-run time indicator, age, transformational leadership, employee well-being, and technological advancement. All VIF values were below the conventional threshold of 10, indicating no evidence of problematic multicollinearity. EMS = emergency medical services; OR = organizational resilience.
